# Juvenile polyposis syndrome in a child with von Willebrand disease: a case report and literature review

**DOI:** 10.3389/fped.2025.1573544

**Published:** 2025-07-02

**Authors:** Yang Yang, Qiong Chen

**Affiliations:** Department of Gastroenterology, Wuhan Children’s Hospital (Wuhan Maternal and Child Healthcare Hospital) Tongji Medical College, Huazhong University of Science & Technology Wuhan, Hubei, China

**Keywords:** juvenile polyposis syndrome, endoscopic polypectomy, complication, post-polypectomy bleeding, von Willebrand disease

## Abstract

**Background:**

Juvenile polyposis syndrome (JPS) is a rare autosomal dominant genetic disorder characterized by multiple gastrointestinal juvenile polyps. Endoscopic polypectomy is the primary therapeutic approach, minor post-polypectomy bleeding is the most common complication. We report an exceptional case of massive hemorrhage (approximately 400 ml) in a child with JPS.The cause of the post-polypectomy bleeding was relatively rare and was ultimately diagnosed as von Willebrand disease (VWD).

**Case presentation:**

A six-year-old girl with JPS and no prior bleeding history underwent endoscopic polypectomy for 11 colorectal polyps.Laboratory tests showed normal platelet count, activated partial thromboplastin time (APTT), prothrombin time (PT), and plasma fibrinogen levels. However, approximately 70 hours after endoscopic polypectomy, she developed hematochezia with significant blood loss (approximately 400 ml). Emergent endoscopic findings did not support technical complications (e.g., clip dislodgement) as the primary etiology of the post-polypectomy hemorrhage.Genetic testing identified a mutation in the von Willebrand factor (VWF) gene [c.1707(exon14)delC, heterozygous], leading to a diagnosis of type 1 von Willebrand disease, which subsequently led to the unexpected post-polypectomy bleeding.

**Conclusion:**

The rare case of juvenile polyposis syndrome with von Willebrand disease in a child underscores the necessity of taking extrinsic gastrointestinal factors into account when delayed post-polypectomy bleeding arises following endoscopic polypectomy. Clinicians ought to be watchful for coagulation disorders, such as VWD, which might be manifested through atypical clinical symptoms. Timely identification of the cause of delayed post-polypectomy bleeding can improve prognosis.

## Introduction

Most colorectal polyps in children are solitary lesions; however, multiple polyps are observed in conditions such as Peutz-Jeghers syndrome (PJS) and juvenile polyposis syndrome (JPS). JPS is an autosomal dominant disorder characterized by hamartomatous gastrointestinal polyps, predominantly affects the colon and rectum, with less frequent involvement of the stomach and small intestine. The number of gastrointestinal polyps varies among patients, ranging from as few as five to over a hundred, depending on the penetrance (1). Patients with JPS may present with clinical manifestations such as hematochezia, anemia, and severe malnutrition. They also have a significantly increased risk of developing colorectal cancer and other related malignancies, leading to a poor prognosis. JPS does not heal spontaneously, and its treatment generally involves polypectomy or organ resection. For small pedunculated polyps, endoscopic cauterization or snare polypectomy should be performed whenever possible. Surgical treatment should be considered for patients with recurrent rectal bleeding, severe anemia, malnutrition, significant complications from polyps, or when endoscopic removal is not feasible (2). Additionally, COX-2 inhibitors and sirolimus are promising therapeutic options for patients with JPS, but they are still in the early stages of research, and their efficacy requires further investigation (3). The main complications after polypectomy under colonoscopy are bleeding, coagulopathy, and perforation, while minor bleeding is the most common complication (4).

Notably, post-polypectomy bleeding due to undiagnosed coagulopathies is underrecognized. VWD, the most prevalent inherited bleeding disorder, which can be inherited in an autosomal dominant or recessive manner. However, its clinical presentation is highly heterogeneous, with bleeding symptoms varying in severity, leading to a high risk of missed or incorrect diagnoses (5). Reports of delayed postoperative significant bleeding after endoscopic polypectomy for JPS in children with VWD are rare. We describe our experience treating a child with JPS and VWD, who experienced post-polypectomy bleeding but ultimately achieved favorable outcomes. We present this case in accordance with the CARE reporting checklist.

## Case presentation

A six-year-old female patient presented to our outpatient clinic on August 7, 2023, with a complaint of intermittent rectal bleeding for over six months. Her stools were soft and tubular, with fresh blood adhering to the surface (minimal volume). Bowel movement frequency ranged from one to three times daily. She occasionally experienced mild periumbilical pain, which did not affect appetite or activity and was unrelated to defecation. No other bleeding symptoms or discomfort were reported, and she had received no prior treatment outside the hospital. Medical or surgical history was unremarkable. Colonoscopy at our hospital's endoscopy center revealed 11 polyps of varying sizes across the colorectum (cecum to sigmoid colon):5 measuring 0.5–1.0 cm, 6 measuring 1.2–1.5 cm. Subsequently, the patient was admitted for further treatment.

### Examination results

The patient's was a well-nourished child with vital stable vital signs, clear consciousness, pink mucous membranes, and no petechiae. Heart and lung examinations were unremarkable. The abdomen was normally contoured and soft to palpation, with mild tenderness near the umbilicus but no rebound tenderness. The spleen and liver were not palpable below the costal margin, and no abdominal masses were detected. The extremities were normal, with a full range of motion, and no abnormalities were observed around the anal region.

Preoperative examinations revealed normal complete blood count and coagulation profiles. Contrast-enhanced computed tomography abdomen and pelvis demonstrated multiple colorectal soft-tissue nodules, consistent with polyps. Given the patient's early disease onset and the presence of multiple gastrointestinal polyps, whole-exome sequencing was recommended to assess recurrence risk and potential malignancy. After adequate preoperative preparations, the patient underwent endoscopic mucosal resection (EMR), snare polypectomy, and hemostatic treatment with titanium clips under general anesthesia. However, 70 hours postoperatively, the patient experienced gastrointestinal hemorrhage, which resolved following emergency endoscopy and blood transfusions. Discharged with dual-specialty follow-up (Gastroenterology & Hematology) for polyp surveillance and bleeding diathesis evaluation.

Prophylactic cryoprecipitate transfusion was administered prior to subsequent polypectomies, with no recurrent hemorrhage.

This study received approval from the Ethics Committee of Wuhan Children's Hospital (No. 2024R105-E01), and informed consent was obtained from the patient's families of the patient for access to clinical data.

### Diagnosis and treatment procedure

**Figure 1:**
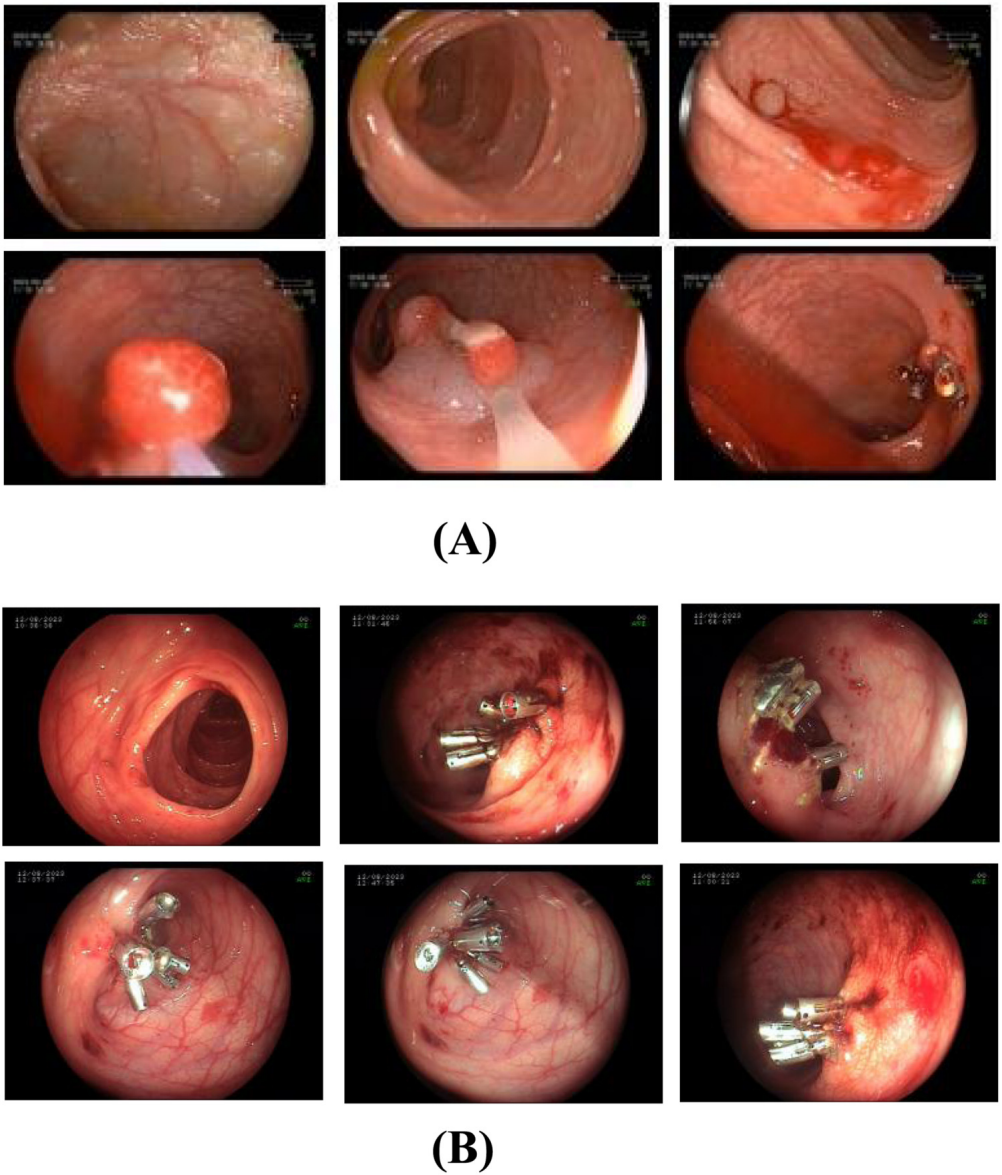
**(A)** first colonoscopy on August 9, 2023. The treatment plan included endoscopic mucosal resection and snare polypectomy for the large and small polyps, respectively. In total, 11 polyps were excised, and 10 titanium clips were used to close the resection sites. Thrombin powder was topically applied for hemostasis. **(B)** Endoscopic view of post-polypectomy bleeding (August 12, 2023). Emergency colonoscopy found approximately 200 ml of dark red blood obstructing visibility in the colon. Active oozing was observed at 5 out of 6 resection sites of large polyps where hemostatic titanium clips had been applied, only 2 of these sites exhibited minor clip dislodgement (1-2 clips lost per site). Persistent bleeding was also evident at resection sites where clips remained securely in place. Additionally, submucosal hemorrhages were identified in areas of normal intestinal mucosa, unrelated to polypectomy wounds. Active bleeding was not observed from the residual polyp stalks after clipping.

Upon admission, the patient was diagnosed with multiple colonic polyps. With parental consent, endoscopic resection was performed under general anesthesia on the third day of hospitalization. Larger polyps were removed using EMR, while smaller ones were excised via snare polypectomy. A total of 11 polyps were resected, with 10 titanium clips applied to close the resection sites. Topical hemostasis was achieved with 1,000 units of lyophilizing thrombin powder (1unit per 100 ml 0.9% sodium chloride). After approximately 10 minutes of observation confirmed no active bleeding, the endoscope was withdrawn (Figure 1A).

Postoperatively, the patient was placed on nothing by mouth (NPO) status with bed rest for 24 hours. Ethylenediamine diaceturate (200 mg/0.2 ml; 2 ml once daily) was administered for hemostasis. A liquid diet commenced 48 hours post-surgery.On postoperative day 4 (August 12, 2023), the patient passed approximately 200 ml of hematochezia but remained asymptomatic. Peripheral blood counts showed normal hemoglobin levels. Emergency colonoscopy revealed approximately 200 ml of dark red blood obscuring colonic visibility. After saline irrigation, examination to the terminal ileum identified no active bleeding source. However, active oozing was observed at 5 of 6 large polyp resection sites previously secured with titanium clips. Minor clip dislodgement (1-2 clips lost) occurred at 2 sites, while bleeding persisted at sites where clips remained intact. Oozing was also noted at one polypectomy site despite secure clip placement. Submucosal hemorrhages were additionally identified in areas of normal mucosa, distinct from polypectomy sites.Nineteen titanium clips were applied to secure 5 actively bleeding sites and 1 non-bleeding site. Subsequent observations confirmed hemostasis. Repeat scope insertion 10 minutes later revealed a clean lumen. Finally, 100 ml of 0.9% sodium chloride solution containing 2,000 units of lyophilizing thrombin powder was instilled into the descending colon (Figure 1B).

Postoperatively, the patient remained NPO but received intravenous fluids, one unit of Rh-positive AB red blood cells, and ceftazidime for injection (1 g twice daily). By day five, hematochezia resolved and a liquid diet was initiated. The patient was discharged in stable condition.Pathological examination of the excised polyps confirmed juvenile polyps, establishing the diagnosis of JPS.

### Final diagnosis

**Figure 2:**
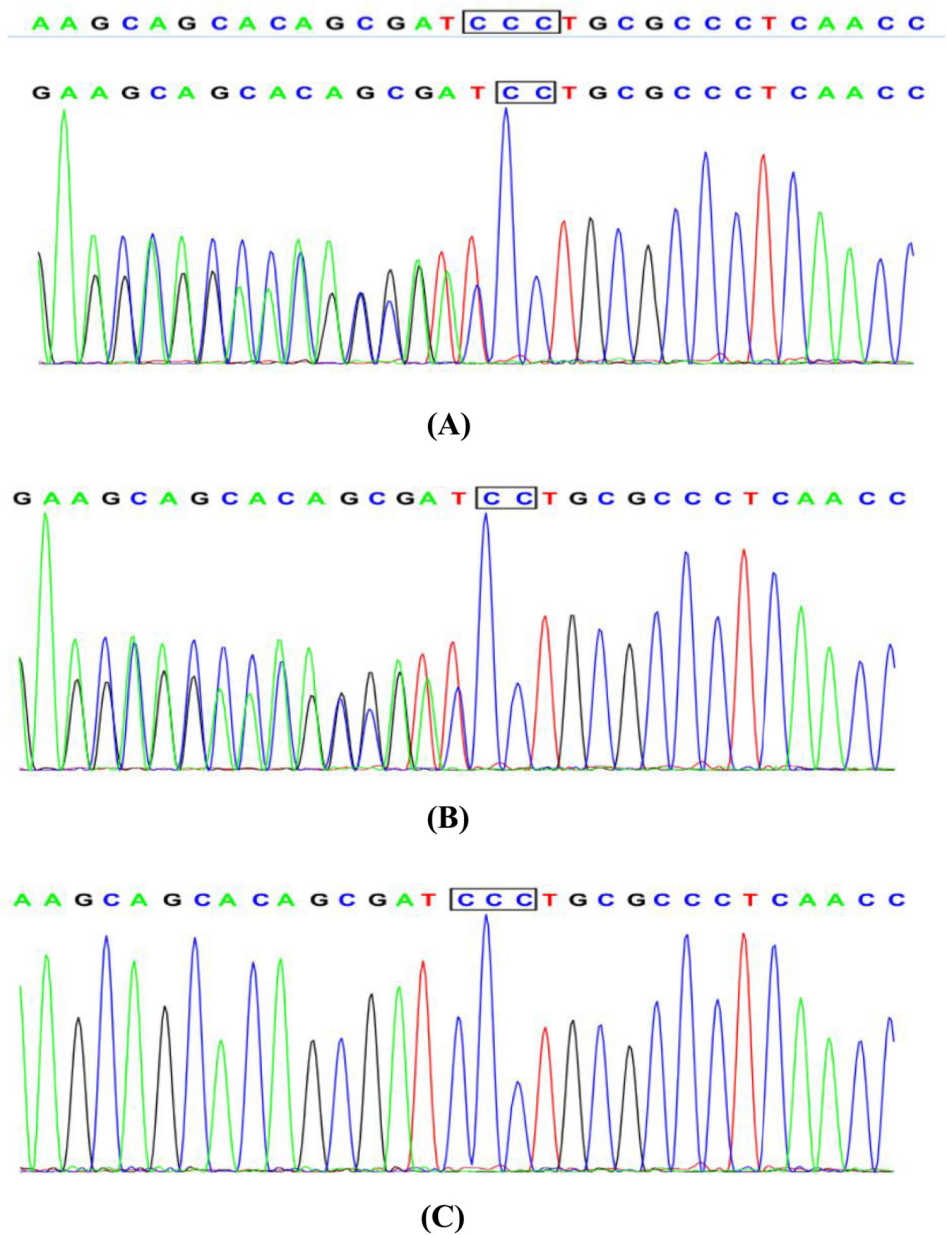
Sanger sequencing chromatogram: **(A)** the patient with juvenile polyposis syndrome and von Willebrand disease: c. 1707(exon14)delC, heterozygous. **(B)** The father of the patient: normal without phenotype, heterozygous. **(C)** The mother of the patient: normal without phenotype, wild type.

**Figure 3:**
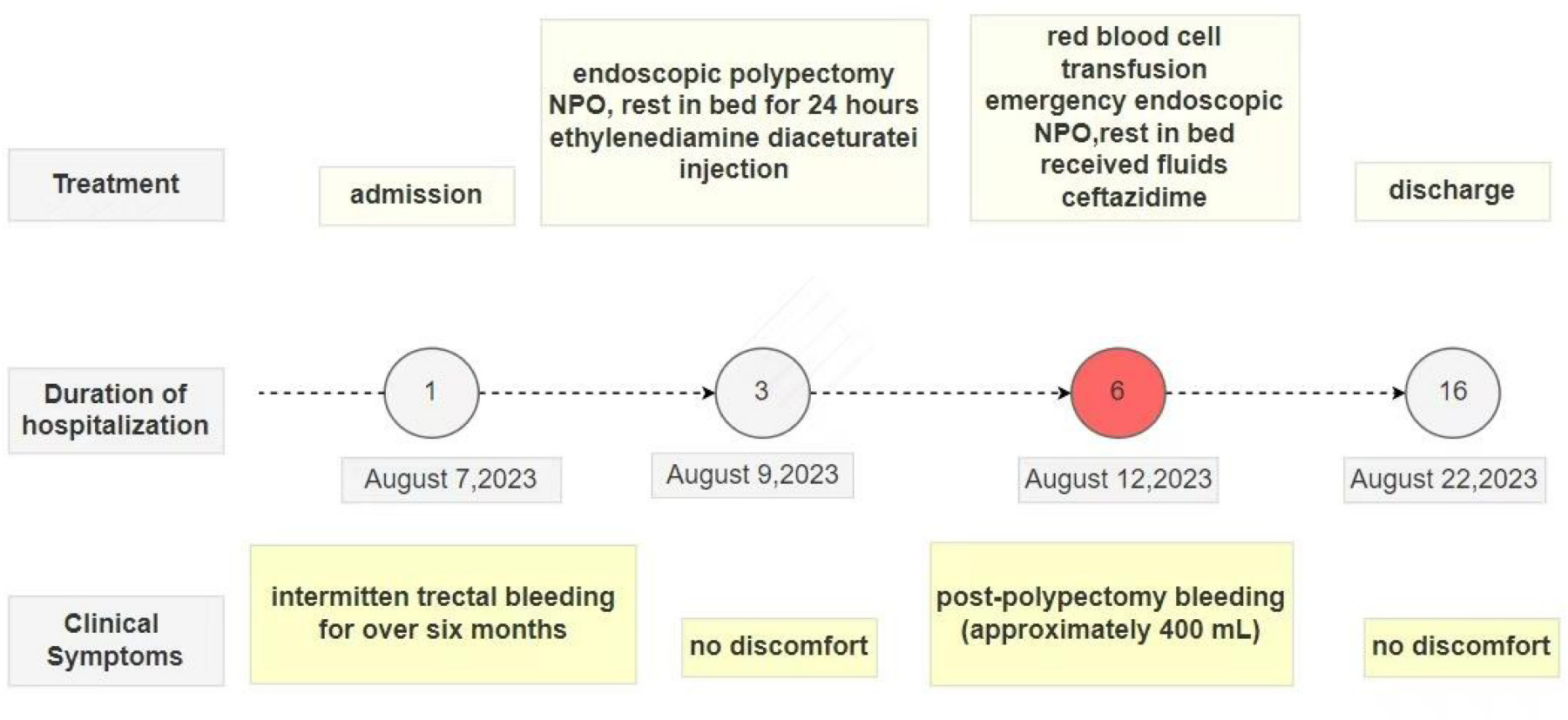
A progress chart presenting the changes in the patient's condition and treatment over time.

The patient's medical history revealed no use of anticoagulants, antiplatelet agents, NSAIDs or corticosteroids, but revealed lifelong mild epistaxis.Familial history was notable for paternal recurrent epistaxis, suggesting hereditary rather than acquired etiology. Prolonged post-polypectomy bleeding prompted genetic testing, which identified a heterozygous VWF gene mutation in both patient and father (Figure 2). Subsequent coagulation studies demonstrated reduced VWF, VWF, and factors IX and X activity, with other factors normal. Although parents declined VWF multimer analysis and 1-deamino-8-D-arginine vasopressin (DDAVP) testing (Table 1A), the collective evidence confirmed type 1 von Willebrand disease.A progress chart presenting the changes in the patient's condition and treatment over time is shown in Figure 3.

**Table 1A:** Complete coagulation panel from February 28, 2024.

Test item	Test result	Reference range
II:C	89.3%	79–131%
V:C	83.3%	62–139%
VII:C	94.9%	50–129%
VIII:C	57.1%	50–150%
IX:C	62.9%↓	65–150%
X:C	66.3%↓	77–131%
XI:C	78.4%	65–150%
XII:C	66.1%	50–159%
VWF:AG	47.8%	66.1–176.3%
VWF activity	43.3%	“Type O”:40.3–125.9%, Other blood types:48.8–163.4%
PT	11.3 s	9.9–128 s
APTT	32.5 s	25.1–36.5 s
INR	1.05	
VWD multimers	Not performed	
DDAVP test	Not performed	

VIII: C, activity of coagulation factor III; IX: C, activity of coagulation factor IX; VWF: Ag, von Willebrand factor antigen.

### Follow-up

At one-year follow-up, the patient reported resolution of hematochezia but persistent mild epistaxis.Repeat colonoscopy and upper gastrointestinal endoscopy demonstrated recurrent colonic polyps and superficial gastritis with fundal erosions.Preoperative VWF activity remained low (consistent with von Willebrand disease). Prophylactic cryoprecipitate (6 units/day × 3 days) was transfused to achieve VWF >50% prior to endoscopic polypectomy (snare resection + titanium clip hemostasis).

Postoperative management included fasting, intravenous fluids, and hemostatic therapy with tranexamic acid (0.39 g IV). After four uneventful days without recurrent bleeding, the patient was discharged. Dual-specialty follow-up (Gastroenterology and Hematology) was arranged.

## Discussion

Juvenile polyposis syndrome is a rare autosomal dominant genetic disorder with an estimated incidence of 1/160, 000–1/100, 000 (6).It increases the risk of gastrointestinal malignancies, with a lifetime risk ranging from 38% to 68% (7, 8). The age of onset in patients with JPS varies widely, with a higher incidence in children. The median age at diagnosis is 18.5 years (9). Family history is present in 50% to 75% of patients (10). Pathogenic variants in the SMAD4 or BMPR1A gene can be detected in 40% to 60% of patients with JPS, and approximately 25% of patients have *de novo* mutations (11, 12). JPS can cause symptoms such as painless rectal bleeding, abdominal pain, moderate to severe anemia, malnutrition, hypoproteinemia, or electrolyte disturbances. It may also be associated with developmental abnormalities, including hydrocephalus, cleft lip and palate, congenital heart disease, cryptorchidism, and malignancy. Currently, the diagnostic criteria for JPS commonly used in clinical practice are those proposed by Jass et al. (6) in 1988:(i) more than five juvenile polyps of the colorectum; and/or (ii) juvenile polyps throughout the gastrointestinal tract; and/or (iii) any number of juvenile polyps with a family history of juvenile polyposis. This case involved a 6-year-old girl with 11 colorectal juvenile polyps (histologically confirmed) and no mucocutaneous pigmentation, fulfilling criterion (i).

Endoscopic polypectomy is the primary treatment for JPS, as it can reduce complications related to polyps and prevent malignant transformation. Minor post-polypectomy bleeding is the most common complication, which can be classified into immediate (intraoperative) and delayed (postoperative) bleeding. Delayed postoperative bleeding is the most common type, typically occurring one to three days after the procedure and often presenting as minor bleeding or positive occult blood. This usually improves with the use of hemostatic agents. In cases of severe delayed bleeding leading to hemorrhagic shock, red blood cell transfusion and emergency endoscopic or surgical intervention may be necessary. Two Chinese single-center studies reported delayed bleeding rates of 3.8% and 4.5%, respectively, following pediatric colorectal polypectomy (13, 14). Postoperative bleeding risk is influenced by three key determinants:(1)patient-specific characteristics(e.g., sex, age, comorbid conditions);(2)polyp morphology (e.g., size and location), and (3)operator experience (15, 16).

Von Willebrand disease is classified into three distinct subtypes(type 1, 2, and 3) with distinct diagnostic criteria and clinical phenotypes, as detailed in Table 1B (5). Type 1 von Willebrand disease, the most prevalent subtype of VWD accounting for approximately 75% of all cases, is inherited in an autosomal dominant pattern. It manifests clinically as mild bleeding tendencies, including menorrhagia during the menstrual cycle and prolonged bleeding following dental extraction or minor surgical procedures. VWD requires individualized treatment based on specific diagnosis, bleeding phenotype, and specific clinical context.Major therapies include use of desmopressin to induce endothelial release of stored VWF and factor VIII and use of VWF concentrates, including both plasma-derived and recombinant products, as well as adjuvant therapies, such as antifibrinolytic tranexamic acid.Targeting specific mutations may further lead to personalized treatments in the future (17). Desmopressin is the first-line treatment for type 1 von Willebrand disease.For those who do not respond, who have contraindication to desmopressin or need prolonged treatment, replacement therapy with FVIII/VWF concentrates is the mainstay of treatment. For children undergoing minor procedures (e.g., polypectomy), consensus guidelines recommend achieving perioperative VWF activity ≥50 IU/dl via DDAVP testing or concentrate titration (18).

**Table 1B:** Classification and clinical manifestations of VWD.

Clinical manifestations	Type 1	Type 2A	Type 2B	Type 2M	Type 2N	Type 3
Inheritance pattern	Autosomal dominant or incompletely dominant	Autosomal dominant or autosomal recessive	Autosomal dominant	Autosomal dominant or autosomal recessive	Mostly autosomal recessive	Autosomal recessive
Bleeding tendency	Mild to moderate	Mostly moderate, individual variation is large	Mostly moderate, individual variation is large	Mostly moderate, individual variation is large	Mostly moderate, individual variation is large	Severe
Pathological features	Partial reduction in VWF quantity	Reduced platelet adhesion	Increased adhesion to platelet GP Ⅰ b	Normal VWF multimers, reduced platelet adhesion	Significantly reduced FⅧ affinity and level	VWF completely absent
VWF:Ag	Reduced	Reduced or normal	Reduced or normal	Reduced or normal	Mostly normal	Absent(<3%)
VWF:RCo	Reduced	Reduced	Reduced	Reduced	Mostly normal	Absent(<3%)
FⅧ:C	Reduced	Reduced or normal	Reduced or normal	Reduced or normal	Significantly reduced	Significantly reduced
VWF:RCo/VWF:Ag ratio	>0.7	<0.7	<0.7	<0.7	>0.7	-
RIPA	Reduced	Reduced	Increased	Reduced	Mostly normal	Absent
VWF multimers	Normal	Abnormal (lack of large and medium—sized multimers)	Abnormal (lack of large molecular multimers)	Normal	Normal	None
DDAVP test	Effective, increased multimers	Partially effective, increased medium—sized multimers	Causes thrombocytopenia	Partially effective, increased multimers	Partially effective, increased multimers	Ineffective

GP Ib, glycoprotein Ib; FⅧ, fibrinogen VIII; VWF:RCo, ristocetin cofactor activity; FⅧ:C, factor VIII procoagulant activity; RIPA, ristocetin-induced platelet aggregation.

This case report describes a 6-year-old child with JPS who underwent endoscopic resection of 11 colorectal polyps. The polyps were removed using a combination of hot biopsy forceps electrocautery and snare-assisted high-frequency electrocoagulation, followed by placement of hemostatic titanium clips at resection sites to prevent delayed bleeding.Unexpectedly, the patient experienced rectal bleeding over 70 hours postoperatively, with an estimated blood loss of 400 ml. Prompt and aggressive hemostatic measures were initiated, and the bleeding cause was sought. An x-ray was done to exclude intestinal perforation. Comprehensive whole-exome sequencing detected a mutation in the VWF gene [c. 1707(exon14)delC, heterozygous], leading to a final diagnosis of VWD. Post-polypectomy, the child had multiple minor gastrointestinal lesions, including lesions where titanium clips remained securely positioned. These lesions, reliant on a normal coagulation system for local blood integrity, bled due to the child's VWD.

Delayed post-polypectomy bleeding in children with JPS and VWD remains poorly documented in current literature. VWD is characterized by highly heterogeneous clinical manifestations and nonspecific bleeding tendencies, leading to frequent underdiagnosis. The present case involved a previously healthy adolescent female with no significant medical/surgical history, premenarchal status, and no prior episodes of menorrhagia. Preoperative assessments including complete blood count and coagulation profiles (PT/APTT) revealed no abnormalities. The patient fulfilled clinical criteria for endoscopic polypectomy based on established JPS diagnostic guidelines.Notably, oozing hemorrhage was endoscopically observed at sites where titanium clips were securely placed, despite optimal clip positioning and absence of mechanical dislodgement.Additionally, submucosal hemorrhages were identified in areas of normal intestinal mucosa, unrelated to polypectomy wounds. Further investigation into the cause of the postoperative bleeding, raising suspicion for congenital coagulation disorders. Whole-exome sequencing confirmed the diagnosis of VWD, which was identified as the primary cause of the postoperative bleeding. Given that JPS requires regular endoscopic follow-up and treatment, prophylactic cryoprecipitate therapy before polypectomy can prevent bleeding caused by coagulation abnormalities at the wound site. This approach reduces patient suffering, minimizes healthcare resource utilization, and could potentially saving lives.

## Conclusion

The aim of this case report is to stress that postoperative rectal bleeding after colorectal polypectomy might also be associated with extrinsic gastrointestinal factors. Clinicians should be mindful of coagulation dysfunction associated with VWD even if clinical symptoms are atypical. Whole-exome sequencing can accurately diagnose congenital coagulation disorders. In such cases, during the perioperative period, it should be considered to prophylactically administer plasma-derived factor VIII concentrates containing VWF or plasma-derived/recombinant VWF products. If conditions are limited, cryoprecipitate or fresh blood can also be used to prevent adverse events like massive postoperative bleeding.

## Data Availability

The raw data supporting the conclusions of this article will be made available by the authors, without undue reservation.
